# Deep-Learning Enabled Atomistic Understanding of Thermomechanical Behaviors and Fracture Mechanisms of High-Entropy Diboride (Hf_0.2_Zr_0.2_Ta_0.2_Ti_0.2_Nb_0.2_)B_2_

**DOI:** 10.3390/ma19132785

**Published:** 2026-07-01

**Authors:** Xu Zhang, Bei Li, Meng Wang, Bo Liu, Ji Zou, Jianjun Li

**Affiliations:** 1 School of Materials Science and Engineering, Research Center for Materials Genome Engineering, Wuhan University of Technology, Wuhan 430070, China; zhxu@whut.edu.cn (X.Z.); wangmeng10330@whut.edu.cn (M.W.); 2 State Key Laboratory of Advanced Technology for Materials Synthesis and Processing, Wuhan University of Technology, Wuhan 430070, China; liubo16@whut.edu.cn; 3 International School of Materials Science and Engineering, Wuhan University of Technology, Wuhan 430070, China; 4 State Key Laboratory of Materials Processing and Die & Mould Technology, Huazhong University of Science and Technology, Wuhan 430074, China; jianjun@hust.edu.cn

**Keywords:** high-entropy diboride, deep-learning potential, molecular dynamics, fracture mechanisms

## Abstract

**Highlights:**

- A deep-learning potential enables large-scale MD of high-entropy diboride.
- Anisotropic loading reveals strong tension–compression asymmetry.
- Strength and ductility degrade by over 45% and 30% between 900 K and 3300 K.
- Tension fails via bond rupture, while compression fails via densification and shearing.

**Abstract:**

High-entropy transition-metal diborides represent a promising class of ultra-high temperature ceramics. However, atomic insights into their high-temperature elastic response, anisotropic deformation, and fracture mechanisms remain elusive. Herein, we perform molecular dynamic simulations to study the thermomechanical behaviors of (Hf_0.2_Zr_0.2_Ta_0.2_Ti_0.2_Nb_0.2_)B_2_ from 900 to 3300 K by developing an ab initio accuracy deep-learning potential. The proposed potential accurately reproduces lattice parameters, equations of state, and elastic constants, in excellent agreement with density functional theory calculations and available experiments, and remains transferable under thermally expanded and compressed states. The simulations reveal anisotropic thermal expansion, with the out-of-plane expansion exceeding the in-plane expansion, together with progressive elastic softening while preserving *C*_11_ > *C*_33_ due to the dominant in-plane B-B bonding network. Furthermore, strain-rate- and temperature-dependent tensile and compressive responses show marked crystallographic anisotropy, tension–compression asymmetry, and severe thermomechanical degradation. Atomic structural evolution demonstrates that tensile fracture is dominated by bond stretching and progressive damage accumulation, whereas compressive failure is attributed to densification- and shear-mediated structural instability. These findings provide an atomistic understanding of the thermomechanical behavior and fracture mechanisms of the prototypical single-phase (Hf_0.2_Zr_0.2_Ta_0.2_Ti_0.2_Nb_0.2_)B_2_ high-entropy diboride, offering valuable insights into the design of ultra-high temperature ceramics under extreme service environments.

## 1. Introduction

Ultra-high temperature ceramics (UHTCs), such as transition-metal diborides, have drawn remarkable attention in numerous applications including hypersonic flight vehicles, rocket propulsion components, and atmospheric re-entry systems, owing to their extremely high melting points (≥3000 K) and excellent thermochemical stability [1,2,3,4,5]. However, their broader utilization can be limited by intrinsic brittleness and pronounced mechanical degradation at elevated temperatures. Recently, the high-entropy strategy, originally developed for metallic alloys, has been extended to ceramic systems, enabling the formation of chemically disordered and entropy-stabilized solid solutions with outstanding properties that exceed those of conventional single-component counterparts [6,7,8]. Many experimental and theoretical studies have shown the potential of high-entropy diborides (HEBs, i.e., (Hf_0.2_Zr_0.2_Ta_0.2_Ti_0.2_Nb_0.2_)B_2_) to serve as promising UHTCs that combine extremely high melting points with improved thermal and mechanical properties [9,10,11,12,13,14,15,16,17]. Despite these advancements, previous studies have primarily concentrated on phase formation, oxidation resistance, and mechanical properties at room or elevated temperatures (≤2600 K), whereas the atomistic origins of anisotropic deformation, strength degradation, and fracture failure at ultra-high temperatures (≥3000 K) remain poorly understood [14,15]. This knowledge gap arises from the chemically disordered nature of HEBs and their coherent complex bonding characteristics, i.e., the covalent B-B interactions, metallic metal–metal (M-M) interactions, and mixed metal-B (M-B) coupling. This specifically creates a heterogeneous bonding network that governs elastic anisotropy, deformation pathways, and temperature-dependent mechanical properties [12,13]. Therefore, it is of vital importance to examine the dynamic mechanical behaviors and associated failure mechanisms of HEBs under ultra-high temperature conditions from an atomic perspective.

Although density functional theory (DFT) offers valuable insights into elasticity, chemical bonding, and idealized deformation pathways [12,18], the intrinsic chemical disorder of HEBs usually requires large supercells that are computationally prohibitive for conventional ab initio calculations. More importantly, dynamic processes relevant to mechanical failure, including thermally activated bond breakage, defect nucleation, and crack initiation, necessitate scalable finite-temperature simulations that standard DFT is unable to access. Contrarily, molecular dynamic (MD) simulation provides a robust framework for investigating large-scale, finite-temperature deformation and fracture mechanisms at an atomistic level [19,20,21]. The predictive capability of classical MD, however, crucially depends on the accuracy and transferability of the underlying interatomic potentials. For chemically complex systems such as HEBs, the development of robust empirical potentials is particularly challenging because of the vast compositional and configurational sampling space. Thus, the state-of-the-art machine-learning interatomic potentials have become a powerful alternative to achieve near quantum accuracy while maintaining the computational efficiency required for large-scale simulations [22,23,24,25,26,27,28]. Recent studies have demonstrated that machine-learning potentials can accurately reproduce the lattice parameters, elastic properties, and melting points of high-entropy ceramics [17,29,30]. However, previous machine-learning-aided MD simulations have mainly focused on the equilibrium thermophysical properties; little attention has yet been devoted to the ultra-high-temperature mechanical response of HEBs under large deformation and fracture-relevant loading conditions.

Therefore, in this work, we at first develop a quantum-level machine-learning interatomic potential for the prototypical HEB, namely (Hf_0.2_Zr_0.2_Ta_0.2_Ti_0.2_Nb_0.2_)B_2_, by using a deep neural network framework and an active-learning strategy. The accuracy of the deep-learning potential is verified by predicting the lattice parameters, elastic constants, and energy vs. volume equations of state (EoS) compared with DFT calculations and experiments. Then, large-scale deep-learning-aided MD (DPMD) simulations are conducted to study temperature-dependent elastic properties and the corresponding dynamic mechanical behaviors. Both tensile and compressive responses at different temperatures up to 3300 K are explored to elucidate the anisotropic deformation characteristics and fracture dynamics. Additionally, the crucial role of the complex bonding network (i.e., the B-B, M-M, and M-B interactions) in the structural evolution during deformation is systematically analyzed. The present work is envisioned to fill the knowledge gap and offer an atomistic understanding of the dynamic mechanical behavior and underlying fracture mechanisms, thereby providing intuitive guidance for designing UHTCs with desirable thermomechanical properties under severe ultra-high-temperature environments.

## 2. Computational Methods

### 2.1. Dataset Preparation

**Figure 1:**
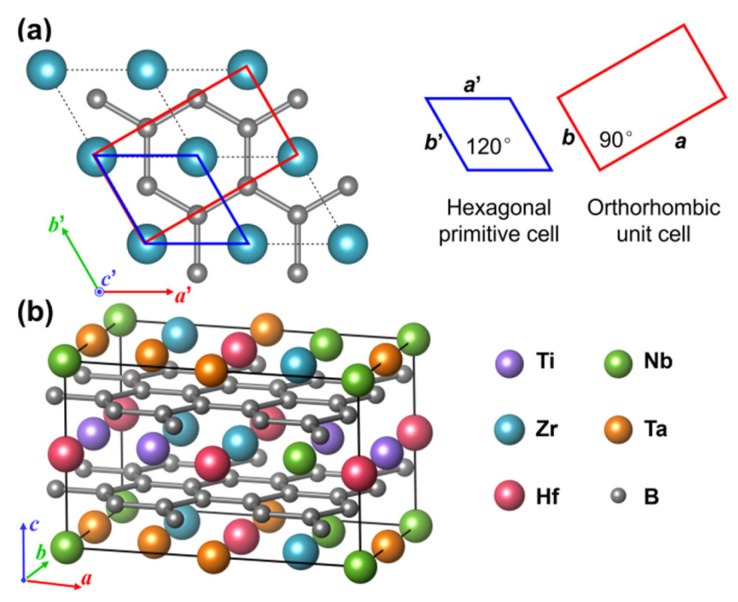
(**a**) Schematic illustration of the transformation between the hexagonal and orthorhombic unit cells, and (**b**) a typical atomic configuration of (Hf_0.2_Zr_0.2_Ta_0.2_Ti_0.2_Nb_0.2_)B_2_. Here, *a*’, *b*’, and *c*’ denote the lattice vectors of the hexagonal primitive cell, whereas *a*, *b*, and *c* denote those of the orthorhombic unit cell.

A comprehensive yet non-redundant training dataset is essential to accurately represent the potential energy surface. The initial configuration of (Hf_0.2_Zr_0.2_Ta_0.2_Ti_0.2_Nb_0.2_)B_2_ was constructed based on the AlB_2_-type hexagonal crystal structure (space group P6/mmm, No. 191) [31], which has been experimentally observed in high-entropy metal diboride ceramics [16,32]. The starting lattice parameters were set to *a* = *b* = 3.108 Å and *c* = 3.348 Å, corresponding to the arithmetic averages of the lattice constants of the constituent binary diborides MB_2_ with M = Hf, Zr, Ta, Ti, or Nb. These averaged values were used solely as initial geometric parameters for generating the chemically disordered configurations. Although the AlB_2_ structure is generally described by using a hexagonal unit cell, an orthorhombic representation was adopted here to eliminate artificial symmetry restrictions and allow for fully unconstrained atomic motion in MD simulations. The orthorhombic unit cell was constructed as a $\sqrt{3}$ × 1 × 1 supercell of the hexagonal sub-cell, in which the *b*-axis is expanded and then rotated by 30° within the basal-plane, while the *b*- and *c*-axes remain unchanged, as depicted in Figure 1a. Figure 1b shows a typical 2 × 2 × 2 orthorhombic supercell containing 48 atoms, which was generated using the special quasi-random structure (SQS) method implemented in the “mcsqs” code of the Alloy Theoretic Automated Toolkit (ATAT) [33,34]. However, achieving an exact equimolar distribution of the five metal atoms is impossible in the 48-atom supercell. Thus, five near-equimolar compositions were used to approximate the target equimolar ratio while maintaining the MB_2_ stoichiometry, i.e., Hf_3/16_Zr_3/16_Ta_3/16_Ti_3/16_Nb_1/4_B_2_, Hf_3/16_Zr_3/16_Ta_3/16_Ti_1/4_Nb_3/16_B_2_, Hf_3/16_Zr_3/16_Ta_1/4_Ti_3/16_Nb_3/16_B_2_, Hf_3/16_Zr_1/4_Ta_3/16_Ti_3/16_Nb_3/16_B_2_, and Hf_1/4_Zr_3/16_Ta_3/16_Ti_3/16_Nb_3/16_B_2_. In this way, a total of 6300 configurations were generated using the SQS method to construct the initial training dataset.

To enhance configurational diversity, each structure was further perturbed via (i) uniform lattice scaling with a scaling factor *λ* of 0.97, 1.00, or 1.03; (ii) random cell deformation with a strain in the range of −3% to +3% applied to each lattice parameter (*a*, *b*, *c*, *α*, *β*, or *γ*); and (iii) random atomic displacements of up to ±0.3 Å along each direction. Then, 18,900 distorted configurations were generated and subsequently subjected to the ab initio MD simulations for 10 steps in the NVT ensemble at 300 K. Such a short dynamic procedure was implemented to eliminate the high-energy artifacts introduced by the artificial distortions, rather than to achieve thermodynamic equilibration.

Furthermore, the Deep Potential Generator (DP-GEN) framework was employed to iteratively expand the distorted structure dataset through an active-learning strategy [35]. Specifically, 100 configurations were selected randomly from the initial 6300 structures with diverse elemental distributions. These configurations were further explored via MD simulations in the NPT ensemble over temperatures from 1600 to 3600 K and pressures from 0 to 1000 bar, to sample thermally activated and stress-driven local environments relevant to high-temperature deformation. Through successive DP-GEN iterations, 3127 candidate configurations were automatically generated and their total energies, atomic forces, and virial tensors were labeled using DFT calculations. This produced a final dataset of 22,027 configurations, from which a total of 1,057,296 local atomic environments were extracted. The dataset preparation workflow is also depicted in Figure S1 in the Supporting Information document. The final dataset was then divided into training, validation, and test subsets containing 18,247 (82.84%), 1890 (8.58%), and 1890 (8.58%) configurations, respectively. As compared to the commonly used proportion of 70–80%, the relatively larger training dataset was adopted to ensure the comprehensive coverage of chemically diverse local environments within the multicomponent (Hf_0.2_Zr_0.2_Ta_0.2_Ti_0.2_Nb_0.2_)B_2_ system.

### 2.2. Training of the Deep-Learning Potential

The deep-learning interatomic potential was trained using the DeepMD-kit framework with the se_atten descriptor, which is specifically designed for multicomponent systems [25,36]. This descriptor uses an embedding network to automatically encode element types, relative atomic coordinates, and local neighbor environments into symmetry-preserving representations. The embedding network consisted of three fully connected layers with 80, 160, and 320 neurons, respectively, while the fitting network contained three layers with each having 160 neurons. The atomic environment descriptors were defined to decay smoothly to zero over the radial cutoff range of 0.2–6.0 Å. To account for the substantially increasing number of neighboring atoms under compressive loading in the following MD simulations, the maximum number of neighbors for each chemical species was conservatively set to be 374 for ensuring reliable interatomic interactions. The model parameters were optimized using an exponentially decaying learning rate, which reduced from 1.0 × 10^−3^ to 3.51 × 10^−8^ over 2 × 10^6^ training steps, with a decay interval of 10,000 steps. The initial weighting factors in the loss function for the energy, force, and virial terms were set to be 0.02, 1000, and 0.02, respectively, and were progressively adjusted to 1.0 during the course of training.

### 2.3. DFT Calculations

All the total energies, atomic forces, and virial tensors were obtained from DFT calculations by using the Vienna ab initio Simulation Package (VASP) [37]. The exchange-correlation effect was treated using the Perdew-Burke-Ernzerhof (PBE) functional within the generalized gradient approximation (GGA), and the interactions between core and valence electrons were described using the projector augmented-wave (PAW) method [38]. A plane-wave basis set with a kinetic energy cutoff of 450 eV was employed. The electronic self-consistency was achieved with an energy convergence criterion of 1.0 × 10^−5^ eV/atom, and all structures were relaxed until the residual atomic forces were below 0.01 eV/Å. The Brillouin zone was sampled using a Monkhorst–Pack scheme with a k-point spacing of 0.05 Å^−1^.

### 2.4. MD Simulations

The MD simulations were performed using the Large-scale Atomic/Molecular Massively Parallel Simulator (LAMMPS) package [39], with interatomic interactions described based on the trained deep-learning potential. The metal atoms were stochastically distributed over equivalent lattice sites between adjacent honeycomb B-B layers to mimic the chemical disorder in (Hf_0.2_Zr_0.2_Ta_0.2_Ti_0.2_Nb_0.2_)B_2_. The temperature and pressure were controlled using the Nosé–Hoover thermostat and barostat with damping constants of 0.1 ps and 0.5 ps, respectively. The equations of motion were integrated using the velocity–Verlet algorithm with a time step of 1.0 fs, and periodic boundary conditions were applied in all three directions.

To endorse the accuracy and transferability of the deep-learning potential, the elastic constants and EoS curves were evaluated in a 2 × 4 × 4 orthorhombic supercell. For the elastic constant calculation, the constant-strain minimization method was employed with small independent strain perturbations up to a magnitude of 2.0%. The application of the strain was performed by uniformly expanding the simulation cell in the direction of the deformation and rescaling the atomic coordinates to fit within the new dimensions. After each increment of the applied strain, the potential energy of the structure was re-minimized whilst keeping the lattice parameters fixed. Then, the elastic constant *C_ij_* was calculated from the second derivative of potential energy (*U*) with respect to strain as $C_{ij}=\frac{1}{V}\frac{\partial^{2}U}{\partial \varepsilon_{i}\partial \varepsilon_{j}}=\partial \sigma_{i}/\partial \varepsilon_{j}$, where *V* is the volume, *ε* is the strain, and *σ* is the stress component. The Young’s modulus (*E*), bulk modulus (*B*), and shear modulus (*G*) were then estimated using the Voigt–Reuss–Hill approximation [40]. For the EoS calculation, the cell volume was uniformly scaled within ±15% of the equilibrium volume, and the corresponding total energies were obtained via the conjugate-gradient minimization.

To investigate the thermomechanical behavior, a 6 × 12 × 12 supercell containing 5184 atoms was first energy-minimized using the conjugate gradient method at 0 K and subsequently equilibrated for 20 ps in the NPT ensemble at 900 K and zero pressure. The system was then heated up to 3300 K at a constant rate of 10 K/ps. Configurations were extracted at 900, 1500, 2100, 2700, and 3300 K, and they were further equilibrated for 40 ps under zero-pressure NPT conditions. The temperature-dependent elastic constants *C_ij_* were at first determined from the linear stress–strain relation over the infinitesimal-strain regime in NVT simulations. A strain amplitude of 2% was adopted to balance systematic and statistical errors, yielding stable estimates of *C_ij_*. To further investigate dynamic mechanical behaviors, the uniaxial tensile and compressive tests were performed at a given temperature (i.e., 2100 K) in the NVT ensemble with strain rates of 0.01, 0.005, 0.001, and 0.0005 ps^−1^, respectively. Periodic boundary conditions were applied in all three directions during the uniaxial deformation tests. Then, a strain rate of 0.001 ps^−1^ (see Section 3.3.2) was chosen for the subsequent tensile and compressive calculations at different temperatures. The stress–strain curves were recorded along the *b*- and *c*-axes to characterize the anisotropic elasticity and deformation behaviors resulting from the AlB_2_-type layered structure in HEBs. Moreover, for each tensile or compressive test, three independent random structures were used to capture configurational fluctuations inherent to the chemically disordered (Hf_0.2_Zr_0.2_Ta_0.2_Ti_0.2_Nb_0.2_)B_2_, and the corresponding standard deviations were marked as the uncertainty bars.

## 3. Results and Discussion

### 3.1. Validation of the Deep-Learning Potential

**Figure 2:**
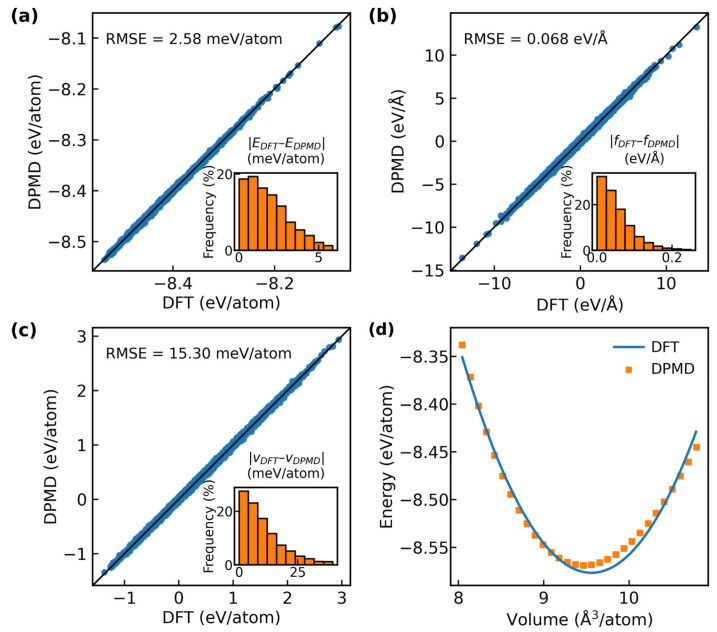
Comparison between DFT and DPMD simulations for (**a**) energies, (**b**) atomic forces, and (**c**) virial tensors in the test dataset, and (**d**) the energy vs. volume EoS of (Hf_0.2_Zr_0.2_Ta_0.2_Ti_0.2_Nb_0.2_)B_2_.

As shown in Figure 2, the DPMD simulations reproduce the DFT data with high fidelity, yielding root-mean-square errors (RMSEs) of 2.58 meV/atom, 0.068 eV/Å, and 15.30 meV/atom for the energy, atomic forces, and virial tensors in the test dataset, respectively. These error levels are markedly lower than those reported in previous machine-learning-aided MD simulations of HEBs and related systems [17,29,30,41,42], indicating that the present deep-learning potential achieves a high degree of accuracy and generalizability without overfitting.

Moreover, the lattice parameters and elastic constants predicted by DPMD are compared with DFT calculations and experimental measurements [16,32], as summarized in Table 1. It is shown that the DPMD results demonstrate excellent agreements with both theoretical and experimental data, with most deviations of less than ~2%. Particularly, the experimentally reported moduli (i.e., *E*, *B*, and *G*) align well with our DPMD-predicted values at 300 K. As compared to the previous work for HEBs [17], the improved accuracy underscores the effectiveness of the employed active-learning training strategy. To examine the transferability of the deep-learning potential, the EoS curve under volumetric deformation at ~0 K was computed using DPMD simulations and was compared to DFT calculations, as shown in Figure 2d. The DPMD-predicted minimum energy is −8.57 eV/atom at a volume of about 9.47 Å^3^/atom, which is in excellent agreement with the DFT result. Notably, albeit the training dataset sampled configurations within a narrow lattice scaling range of 0.97–1.03, the deep-learning potential can accurately reproduce the EoS over a substantially broader volumetric range, corresponding to the lattice scaling of 0.85–1.15. This endorses the scalability and capability of the deep-learning potential model in describing (Hf_0.2_Zr_0.2_Ta_0.2_Ti_0.2_Nb_0.2_)B_2_ under deformed (i.e., expanded or compressed) structural states well.

To further assess the applicability of the deep-learning potential for mechanical deformation and fracture-relevant states, additional AIMD calculations of uniaxial tension and compression along the *b*- and *c*-axes at 900 and 2100 K were performed and compared with DPMD. The calculation details are illustrated in the Supplementary Note S1. As shown in Figure S2, DPMD effectively reproduces the AIMD stress–strain curves across all loading modes, including the initial build-up, the peak stress prior to structural instability, and the subsequent stress relaxation. This agreement confirms that the deep-learning potential accurately describes the orientation- and loading-mode-dependent stress evolution under thermal conditions. However, slight deviations occur, particularly near the instability region, primarily due to the sensitivity of the stress response to instantaneous atomic configurations and thermal fluctuations. Despite this, DPMD successfully captures the overall deformation trend and the crucial instability behavior observed in AIMD, endorsing its applicability to the deformed states investigated in this work.

### 3.2. Temperature-Dependent Thermal Properties

**Figure 3:**
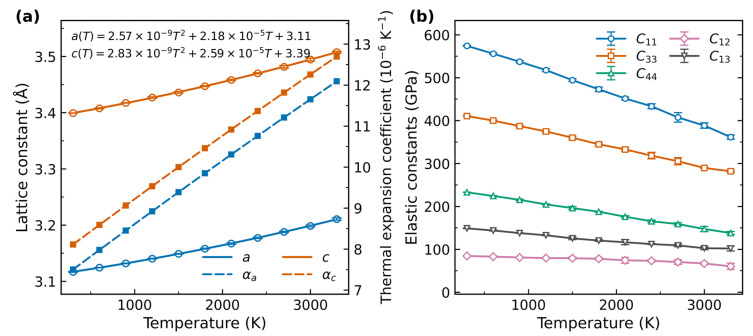
Temperature-dependent (**a**) lattice parameters and thermal expansion coefficients, and (**b**) elastic constants of (Hf_0.2_Zr_0.2_Ta_0.2_Ti_0.2_Nb_0.2_)B_2_.

After validating the deep-learning potential against DFT calculations and available experimental data, the DPMD simulations were further used to predict the finite-temperature thermophysical and elastic responses of (Hf_0.2_Zr_0.2_Ta_0.2_Ti_0.2_Nb_0.2_)B_2_. Figure 3a shows the temperature-dependent lattice parameters and linear thermal expansion coefficients. Both the in-plane lattice parameter and the out-of-plane *c*-axis parameter increase monotonically with temperature, indicating continuous thermal expansion of the AlB_2_-type layered structure. Notably, the out-of-plane thermal expansion coefficient *α_c_* remains higher than the in-plane coefficient *α_a_* over the whole temperature range. This anisotropic expansion is consistent with the bonding characteristics of high-entropy diborides: the strong covalent B-B interactions within the basal boron network suppress in-plane expansion, whereas the relatively weaker mixed M-B coupling and metallic M-M interactions allow more pronounced expansion along the out-of-plane *c*-axis. This trend also supports the reliability of the present high-temperature DPMD predictions. Dai et al. reported the temperature-dependent thermal and elastic properties of high-entropy diborides mainly up to 2400 °C [17]. Their predicted *α_a_* > *α_c_* trend, however, is difficult to reconcile with the conventional bonding picture of AlB_2_-type diborides, in which strong in-plane B-B covalent interactions are expected to constrain basal-plane expansion more effectively than the out-of-plane M-B/M-M interactions. In contrast, the present active-learning strategy explicitly samples high-temperature and compressed configurations, enabling the deep-learning potential to remain stable and transferable up to 3300 K. The obtained *α_c_* > *α_a_* relation is therefore more compatible with the structural chemistry expected from strong in-plane B-B covalent bonding and comparatively weaker out-of-plane interactions.

The temperature-dependent elastic constants are further summarized in Figure 3b. All independent elastic constants decrease progressively with an increasing temperature, demonstrating thermomechanical softening of the (Hf_0.2_Zr_0.2_Ta_0.2_Ti_0.2_Nb_0.2_)B_2_ lattice. Nevertheless, the relative magnitude of the elastic constants remains unchanged throughout the entire temperature range, following *C*_11_ > *C*_33_ > *C*_44_ > *C*_13_ > *C*_12_. Specifically, *C*_11_ and *C*_33_, which characterize the axial stiffness along the in-plane *b*-axis and out-of-plane *c*-axis, respectively, decrease from approximately 571 and 410 GPa at 300 K to about 410 and 292 GPa at 3300 K. The consistently larger *C*_11_ than *C*_33_ indicates that the in-plane direction retains higher resistance to normal deformation, again reflecting the dominant role of the covalent B-B network in the basal plane. The persistent difference between *C*_11_ and *C*_33_ further confirms that the elastic anisotropy is preserved even under severe thermal excitation. Meanwhile, *C*_44_, denoting the shear resistance on the *bc* plane, exhibits a notable decrease (233 to 165 GPa), and the lateral constants *C*_12_ and *C*_13_ reduce by around 36 and 63 GPa, reflecting a diminished transverse coupling response to *b*-axis strain. Moreover, the gradual decrease in *C*_11_, *C*_33_, *C*_44_, and *C*_66_ (*C*_66_ = (*C*_11_ − *C*_12_)/2) demonstrates the distinct temperature-dependent softening effects on the load-bearing capability under normal- and shear-stress conditions, which can promote lattice distortion, strain localization, and damage accumulation (e.g., void nucleation, coalescence, and micro-cracking) at elevated temperatures.

### 3.3. Tensile and Compressive Deformation

Although the elastic constants offer a fundamental measurement of the resistance to infinitesimal deformation, they fail to capture rate-dependent effects, energy dissipation, microstructural evolution, and failure processes under realistic operating conditions (e.g., high-strain-rate loading, cyclic thermal treatment, and vibrational excitations). Therefore, dynamic mechanical analyses are performed by examining the uniaxial tensile and compressive deformation tests, so as to gain deeper insight into the elastic/brittle response and atomistic fracture mechanisms that govern the structural integrity of (Hf_0.2_Zr_0.2_Ta_0.2_Ti_0.2_Nb_0.2_)B_2_ under severe conditions. It is noted that the applied strain rates in DPMD are much higher than those in conventional experimental tests. This discrepancy arises inevitably from the intrinsic spatiotemporal-scale limitation of MD simulations. Accordingly, the following stress–strain curves would represent the idealized, high-rate atomistic response of single crystals, providing mechanistic insight rather than direct quantitative predictions of experimental observation.

#### 3.3.1. Effect of Strain Rate

**Figure 4:**
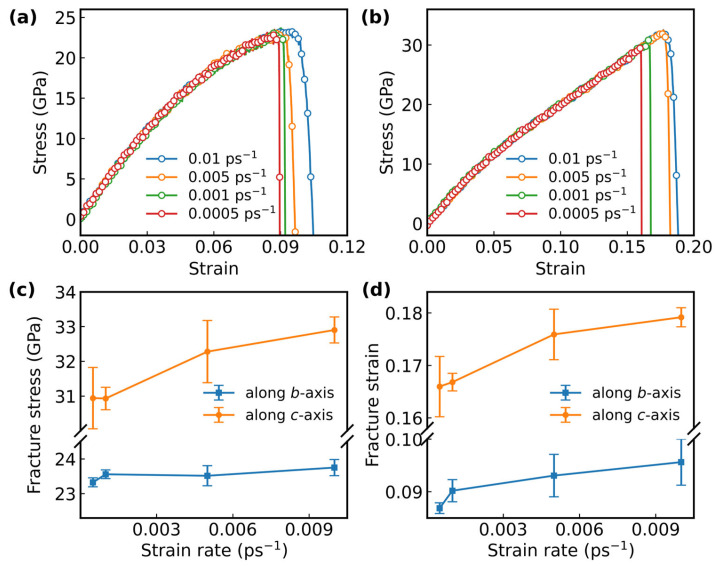
Uniaxial tensile stress–strain curves of (Hf_0.2_Zr_0.2_Ta_0.2_Ti_0.2_Nb_0.2_)B_2_ at 2100 K with different strain rates along the (**a**) *b*-axis and (**b**) *c*-axis. The corresponding fracture stress (*σ_f_*) and fracture strain (*ε_f_*) as functions of strain rate are shown in (**c**) and (**d**), respectively.

During deformation, the strain rate plays a vital role in atomic structural relaxation and defect evolution. The effect of the strain rate on the tensile deformation along the *b*- and *c*-axes is first examined and is shown in Figure 4 for (Hf_0.2_Zr_0.2_Ta_0.2_Ti_0.2_Nb_0.2_)B_2_. For all strain rates, the tensile stress–strain curve is initially linear at low strains, followed by a nonlinear elastic regime, and finally a sudden catastrophic fracture without stable plastic flow, indicating the typical brittle rupture characteristics. Intriguingly, the tensile deformation exhibits pronounced crystallographic anisotropy; namely, the deformation along the *b*-axis shows much lower fracture stresses *σ_f_* (22.6–23.4 GPa) and fracture strains *ε_f_* (0.089–0.096) than those along the *c*-axis (29.5–32.3 GPa and 0.166–0.179). This tensile anisotropy arises mainly from the direction-dependent bonding features inherent to the AlB_2_-type layered structure, where the metal atoms are alternatively separated by the covalently bonded boron honeycomb sheet in the basal-plane. In this manner, the tension along the *b*-axis is governed by the in-plane covalent B-B bonding network and its coupling with neighboring metal atoms, whereas the deformation along the *c*-axis primarily induces the stretching of the interlayer M-B linkage, leading to distinct effective loading paths and cleavage planes. With the increasing strain rate, *ε_f_* increases slightly and *σ_f_* shifts to a relatively higher value for both orientations (Figure 4d). Specifically, the *σ_f_* rises by 4–9% as the strain rate increases from 0.0005 to 0.01 ps^−1^ (i.e., 5 × 10^8^ to 1 × 10^10^ s^−1^), indicating a notable strain-rate strengthening effect. However, these ultrahigh strain rates are inherent to atomistic MD simulations, which are explicitly designed to elucidate qualitative atomistic trends and ideal strength limits. Thus, under higher strain rates, the local structural rearrangement, along with micro-crack initiation and growth, could have insufficient time to evolve, resulting in the premature failure occurring at the relatively higher *σ_f_* and *ε_f_*.

**Figure 5:**
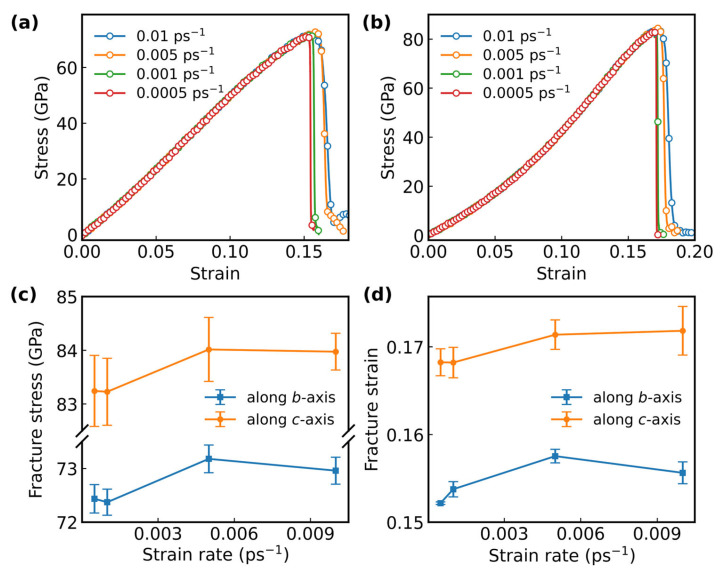
Uniaxial compressive stress–strain curves of (Hf_0.2_Zr_0.2_Ta_0.2_Ti_0.2_Nb_0.2_)B_2_ at 2100 K with different strain rates along the (**a**) *b*-axis and (**b**) *c*-axis. The corresponding *σ_f_* and *ε_f_*, as functions of the strain rate, are shown in (**c**) and (**d**), respectively.

Analogously, Figure 5 shows the compressive stress–strain curves over the same strain rate range. During compression, the stress increases monotonically with the strain and reaches a maximum value followed by abrupt structural instability. Unlike the tensile loading, the stress–strain curves at high strain rates do not drop to zero after fracture (Figure 5a,b), signifying the residual load-bearing capacity associated with densification and shear-dominated load transfer. From Figure 5c, the *σ_f_* values along the *b*- and *c*-axes are around 71.1–72.8 GPa and 82.6–84.3 GPa, respectively, which are ~2.5–3.1 times higher than those for tensile deformation (Figure 4c), demonstrating a pronounced loading mode (tension vs. compression) asymmetry. In addition, both *σ_f_* and *ε_f_* vary only slightly with an increasing strain rate in Figure 5c,d, meaning that the compressive response shows a much weaker strain-rate sensitivity than the tensile response. Given that the strain-rate effect weakens toward the lower bound of the simulated rate window for both tensile and compressive tests, an appropriate strain rate (0.001 ps^−1^) was employed during the following mechanical deformation calculations to improve computational efficiency.

#### 3.3.2. Effect of Elevated Temperature

**Figure 6:**
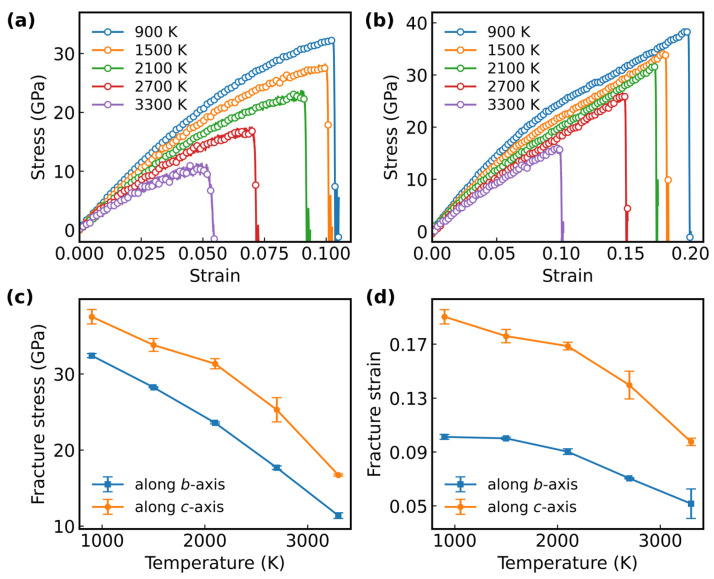
Uniaxial tensile stress–strain curves of (Hf_0.2_Zr_0.2_Ta_0.2_Ti_0.2_Nb_0.2_)B_2_ at a strain rate of 0.001 ps^−1^ with different temperatures along the (**a**) *b*-axis and (**b**) *c*-axis. The corresponding *σ_f_* and *ε_f_* as functions of temperatures are shown in (**c**) and (**d**), respectively.

The load-bearing capacity of HEBs could be substantially deteriorated at elevated temperatures, due to thermally intensified atomic bonding instability. To show the thermal effect, the tensile and compressive deformation along the *b*- and *c*-axes was examined over a temperature range of 900–3300 K for (Hf_0.2_Zr_0.2_Ta_0.2_Ti_0.2_Nb_0.2_)B_2_. In both directions, the tensile deformation proceeds through the three stages at all the temperatures, namely, the initial linear elastic regime, subsequent nonlinear elastic regime, and final fracture failure. However, the variations in *σ_f_* and *ε_f_* are directionally dependent. As seen from Figure 6a, along the *b*-axis, the *σ_f_* reaches ~32.3 GPa at 900 K, with a corresponding *ε_f_* of ~0.10, and then decreases monotonically with the temperature, i.e., *σ_f_* of 11.2 GPa with *ε_f_* of 0.05 at 3300 K. This continuous reduction demonstrates that the thermal treatment suppresses the tensile strength and admissible strain window simultaneously. Meanwhile, the deformation along the *c*-axis consistently exhibits higher tensile resistance and delayed fracture, as seen in Figure 6b. Specifically, *σ_f_* decreases from 38.3 GPa at 900 K to 16.3 GPa at 3300 K, and *ε_f_* declines from 0.20 to 0.10. The temperature dependences of the *σ_f_* and *ε_f_* are further summarized in Figure 6c,d. From 900 to 3300 K, *σ_f_* decreases by around 65% and 57% along the *b*- and *c*-axes, respectively, and *ε_f_* reduces by ~50% in both directions. This confers that the thermally activated atomic vibrations considerably destabilize both the in-plane B-B/M-M bonding and the out-of-plane M-B interactions (Movies S1 and S2). This facilitates earlier bond rupture and stronger strain localization under tensile stresses, while the more pronounced deterioration along the *b*-axis is consistent with the stronger temperature-induced softening of the basal-plane axial stiffness represented by *C*_11_/*C*_22_ (Figure 3b).

**Figure 7:**
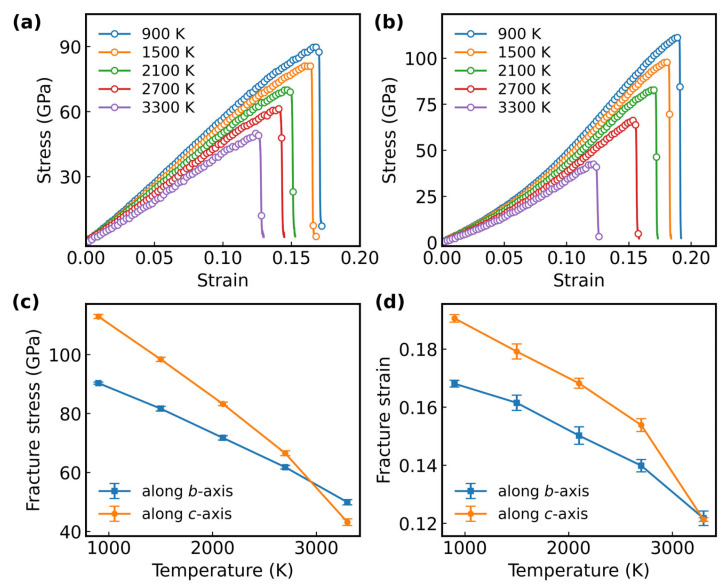
Uniaxial compressive stress–strain curves of (Hf_0.2_Zr_0.2_Ta_0.2_Ti_0.2_Nb_0.2_)B_2_ at a strain rate of 0.001 ps^−1^ with different temperatures along the (**a**) *b*-axis and (**b**) *c*-axis. The corresponding *σ_f_* and *ε_f_*, as functions of temperatures, are shown in (**c**) and (**d**), respectively.

Accordingly, the compressive stress–strain curves in Figure 7 follow the same overall trend of monotonic thermal degradation, although the fracture stresses are substantially higher than those under tension. As plotted in Figure 7c and Figure 7d, *σ_f_* decreases from 90.4 GPa at 900 K to 49.7 GPa by 45% at 3300 K, along with the *ε_f_* shrinking from 0.17 to 0.12 by ~30% along the *b*-axis, whereas the corresponding values reduce from 112.4 to 43.4 GPa by ~61% for *σ_f_* and from 0.19 to 0.12 by ~37% for *ε_f_* along the *c*-axis, respectively. The much larger reduction in *σ_f_* and *ε_f_* along the *c*-axis clearly signifies a more severe softening effect at elevated temperatures, which even leads to higher in-plane resistance to compression at 3300 K, i.e., a larger *σ_f_* along the *b*-axis than that along the *c*-axis. Albeit experimental data on uniaxial tensile and compressive deformation remains scarce, the monotonic reduction in the predicted *σ_f_* and *ε_f_* reflects the intrinsic thermal weakening of the defect-free lattice at elevated temperatures.

#### 3.3.3. Evolution of Structural Deformation

To elucidate the mechanisms governing deformation and failure at elevated temperatures in (Hf_0.2_Zr_0.2_Ta_0.2_Ti_0.2_Nb_0.2_)B_2_, we analyzed the evolution of structural deformation at a given temperature, i.e., 2100 K, where the tensile and compressive stress–strain curves show resemblance with the others over the investigated strain rates and temperatures. Additionally, three structural parameters have been defined to reveal the defect evolution, i.e., the fractions of B atoms (*f*_B-B_ and *f*_B-M_) with reduced B-B and B-M coordination depicting the local bond-network degradation and the normalized largest void-cluster volume ($V_{max}^{void}/V_{box}$) quantifying the extent of crack-like damage regions [43,44,45,46]. The calculation details of these parameters can be found in the Supplementary Note S2.

**Figure 8:**
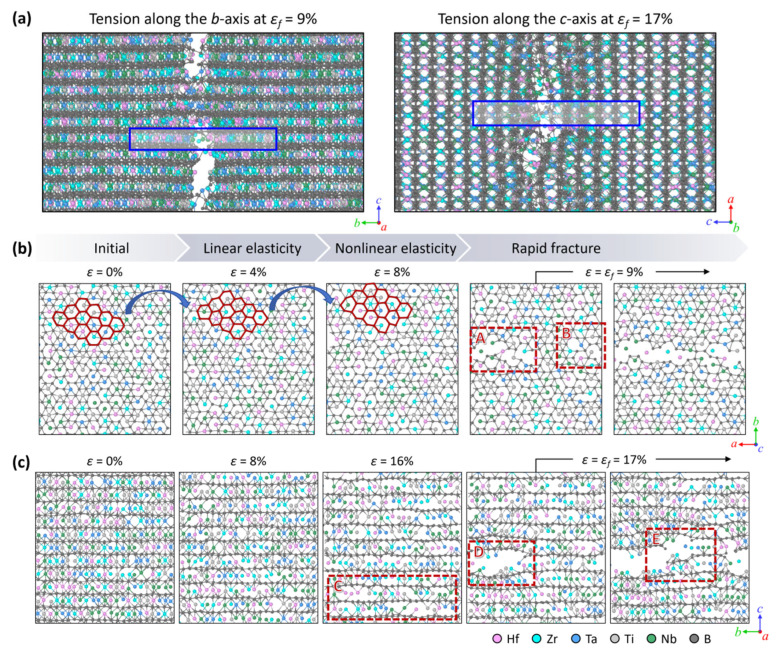
Atomic snapshots of (Hf_0.2_Zr_0.2_Ta_0.2_Ti_0.2_Nb_0.2_)B_2_ under tensile loading with a strain rate of 0.001 ps^−1^ at 2100 K: (**a**) full snapshots of the sample upon fracture and the corresponding projected atomic structural evolution of the slices (denoted by the blue boxes in (**a**)) along the (**b**) *b*-axis and (**c**) *c*-axis, respectively. The arrows indicate the progression of tensile deformation and local structural evolution, while the capital letters A–E denote representative regions highlighted by red dashed boxes for structural analysis. The red solid lines indicate the local B-B network structures.

**Figure 9:**
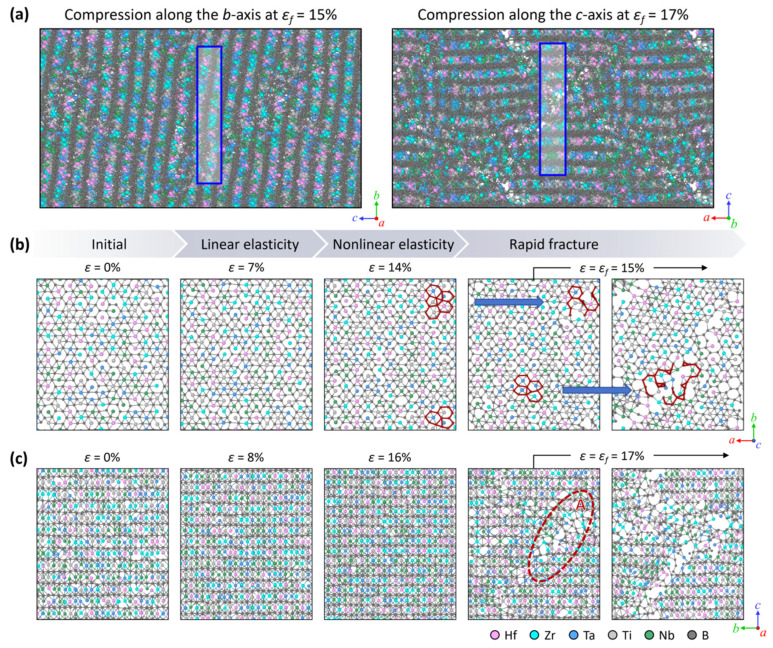
Atomic snapshots of (Hf_0.2_Zr_0.2_Ta_0.2_Ti_0.2_Nb_0.2_)B_2_ under compressive loading with a strain rate of 0.001 ps^−1^ at 2100 K: (**a**) full snapshots of the sample upon fracture and the corresponding projected atomic structural evolution of the slices (denoted by the blue boxes in (**a**)) along the (**b**) *b*-axis and (**c**) *c*-axis, respectively. The arrows indicate the progression of applied strain and local structural deformation, while the capital letter and red dashed lines denote representative regions highlighted for structural analysis. The red solid lines indicate the local B-B network structures.

The full fracture snapshots in Figure 8a and Figure 9a show the pronounced anisotropy of damage evolution and the final failure morphology under tensile and compressive loading. Under tension along the *b*-axis (Figure 8b), the projected atomic configuration remains largely intact from the initial state to the strain *ε* of 4%, suggesting that the deformation is initially sustained via elastic elongation of the lattice. With increasing *ε*, the B layer starts to undergo progressive distortion, showing the continuous breakage and formation of M-B bonds due to thermal fluctuations, as highlighted by the red-marked evolution of the B-B network in Figure 8b. As *ε* approaches 8%, these distortions intensify and lead to the formation of multiple nano-voids, as exemplified by the regions A and B. When these nano-voids interconnect to form a crack-like damage band spanning the entire layer, the structural integrity deteriorates rapidly, giving rise to the brittle fracture occurring at the critical strain *ε_f_* of 9%. Particularly, the fraction of B atoms with reduced B-B coordination *f*_B-B_ increases steadily prior to failure, peaking at 0.255 near *ε* of 9%, which indicates the progressive degradation of the intralayer B-B network (Figure S3a). However, immediately after the stress drop, *f*_B-B_ starts to decrease rather than increase monotonically. This decrease does not indicate the structural recovery. Instead, the rapid unloading following crack formation localizes damage within a connected void region, while the remaining B-rich regions partially relax, restoring some elongated B-B distances back within the coordination cutoff. Also, the largest void cluster emerges after failure and expands to $V_{\max}^{\mathrm{void}}/V_{\mathrm{box}}$ = 0.084, confirming the coalescence of nano-voids into a crack-like damage band (Figure S3b). Therefore, the tensile failure along the *b*-axis can be attributed to a localized damage-propagation mechanism dominated by the stretching-induced rupture of intralayer B-B bonds.

In contrast, under tension along the *c*-axis (Figure 8c), the (Hf_0.2_Zr_0.2_Ta_0.2_Ti_0.2_Nb_0.2_)B_2_ exhibits a different M-B coordination-mediated failure mode. Initially, the atomic configuration remains roughly ordered at *ε* ≤ 8%, indicating that the deformation is initially compensated by the atomic bond stretching with limited local distortion. As the strain increases to 16%, the expanding interlayer spacing progressively weakens the interlayer M-B coordination, resulting in the formation of nanoscale low-density regions, as depicted by the region C. These incipient defect regions preferentially nucleate in the areas with severe lattice distortion and chemical heterogeneity, corresponding to the non-uniform M-B bond network with varying local elastic responses that are intrinsic to the high-entropy solid solution [47]. More intriguingly, beyond 16%, one of the low-density regions quickly develops into a larger damage region D in the interior, which extends approximately along the layered structural plane. With further deformation, this enlarged region induces the rupture of intralayer B-B bonds (highlighted in the region E) and finally evolves into a continuous crack band at *ε_f_* of 17%. Accordingly, the fraction of under-coordinated B atoms rises rapidly near fracture, with *f*_B-B_ and *f*_B-M_ reaching 0.043 and 0.113 at *ε* of ~18%, respectively, concurrent with an increase in $V_{\max}^{\mathrm{void}}/V_{\mathrm{box}}$ to 0.114 (Figure S3). Therefore, as compared to the tension along the *b*-axis, where the fracture is characterized mainly by the stretching-activated rupture of intralayer B-B bonds, the tensile failure along the *c*-axis is rather cooperatively governed by the weakening and breakage of M-B bonds and the subsequent rupture of B-B bonds.

Furthermore, the corresponding structural evolution under compression is shown in Figure 9. It is worth noting that the applied compressive strain can partially offset the thermally induced bond elongation, thereby preserving a larger geometrical adjustment space before entering the instability regime dominated by the strong short-range repulsion. Along the *b*-axis (Figure 9b), the configuration remains relatively homogeneous from the initial state up to *ε* of 7%, whereas at *ε* = 14%, the B-B network has experienced severe bond contraction and in-plane twisting and flattening under both axial compression and basal-plane (*ab* plane) deformation. Consequently, the B hexagonal rings exhibit a markedly higher content of distortion and stacking, some of which are even compressed into pentagon- and triangle-like shapes, leading to the topological reconstruction of the B-B network, as highlighted by the red-marked evolution in Figure 9b. Upon further loading, these defective regions become more compacted, promoting the rupture of B-B and M-B bonds. Once the critical degree of disruption is reached, the rapid structural instability develops, giving rise to the final failure at *ε_f_* of 15%, as depicted by the sharp increase in *f*_B-B_, *f*_B-M_, and $V_{\max}^{\mathrm{void}}/V_{\mathrm{box}}$ in Figure S3. The compression along the *b*-axis is thus dominated by the progressive collapse and topological reconstruction of the B-B network, along with localized M-B bond breaking and crack-plane formation.

Contrarily, under compression along the *c*-axis (Figure 9c), the layered configuration is retained up to *ε* of 16%, while the interlayer spacing decreases gradually with an increase in the local packing density. This shows that the initial compressive response is primarily depicted by the structural densification. In addition to the axial compression, the basal-plane shear further transfers the compaction into in-plane shear deformation. Under the combined effect of normal densification and shear deformation, the B networks in different layers are progressively distorted and undergo B-B bond rupture. When *ε* > 16%, the local lattice distortion becomes increasingly severe and disordered regions begin to nucleate and evolve within the compacted structure, as illustrated by the region A in Figure 9c. With more deformation, these regions continue to grow and even interconnect, ultimately leading to a dominant, full fracture path responsible for the structural instability at *ε_f_* of 17%, where *f*_B-B_ and *f*_B-M_ increase abruptly to 0.276 and 0.419, respectively (Figure S3a). This clearly indicates that the compressive failure along the *c*-axis is a result of the B-B bond rupture and crack propagation through B-B network and M-B linkage, induced by the interlayer compaction and the in-plane shear deformation.

## 4. Conclusions

In this work, we have developed a quantum-level deep-learning interatomic potential for HEB ceramics, i.e., (Hf_0.2_Zr_0.2_Ta_0.2_Ti_0.2_Nb_0.2_)B_2_. Then, large-scale DPMD simulations were conducted to explore the temperature-dependent elastic properties and fracture dynamics up to 3300 K. The developed deep-learning potential shows excellent accuracy and transferability in predicting the lattice parameters, EoS curve, and elastic constants, in good agreement with DFT calculations and available experimental data. The MD simulations reveal that the elastic constants decrease almost linearly with temperature, whereas their intrinsic anisotropic relation is maintained throughout the investigated temperature range. Under the uniaxial loading along the *b*- and *c*-axes, the (Hf_0.2_Zr_0.2_Ta_0.2_Ti_0.2_Nb_0.2_)B_2_ exhibits pronounced crystallographic anisotropy and strong tension–compression asymmetry. In particular, the loading along the *c*-axis generally exhibits higher *σ_f_* and *ε_f_* than those along the *b*-axis under both tension and compression, which decline by ~45–65% and 30–50% as the temperature rises from 900 to 3300 K, indicating pronounced thermomechanical degradation. Moreover, the microstructural evolution reveals that the fracture mechanisms governing the thermomechanical behaviors in the (Hf_0.2_Zr_0.2_Ta_0.2_Ti_0.2_Nb_0.2_)B_2_ are complicated and are a synergistic evolution of bond rupture, local atomic rearrangement, and structural disordering. Specifically, the brittle fracture under tension is governed by bond stretching and progressive damage accumulation, while the failure under compression is primarily characterized by densification- and shear-mediated structural instability. The findings are thus of particular value for understanding and designing advanced (Hf_0.2_Zr_0.2_Ta_0.2_Ti_0.2_Nb_0.2_)B_2_-related UHTCs with desirable thermomechanical performances under extremely-high temperature conditions.

## Figures and Tables

**Table 1:** Comparison of lattice parameters and elastic properties obtained from DPMD simulations, DFT calculations, and experiments.

	DPMD (0 K)	DFT	DPMD (300 K)	Experiments
*a* (Å)	3.119	3.115	3.117	3.1062 [32]
*c* (Å)	3.386	3.394	3.399	3.3755 [32]
*C*_11_ (GPa)	596.46	597.75	572.84	
*C*_33_ (GPa)	407.35	405.20	411.68	
*C*_44_ (GPa)	240.24	246.78	231.80	
*C*_12_ (GPa)	85.55	99.77	84.11	
*C*_13_ (GPa)	148.70	149.86	149.08	
*E* (GPa)	531.17	539.43	506.19	508.53 [16]
*B* (GPa)	261.60	263.95	258.01	259.86 [16]
*G* (GPa)	228.64	232.64	216.04	216.61 [16]

## Data Availability

The original contributions presented in this study are included in the article/Supplementary Materials. Further inquiries can be directed to the corresponding authors.

## Supplementary Materials

The following supporting information can be downloaded at: https://www.mdpi.com/article/10.3390/ma19132785/s1, Note S1: AIMD-DPMD comparison of uniaxial tension and compression; Note S2: Calculation of the fractions of under-coordinated B atoms and the normalized largest void-cluster volume; Figure S1: Workflow of dataset preparation for the deep-learning potential; Figure S2: Comparison of stress-strain curves under different loading modes; Figure S3: Evolution of the structural parameters as a function of strain during tensile and compressive loading along the *b*- and *c*-axes; Figure S4: Radial distribution function profiles of the starting, undeformed structure; Movie S1: Local structural evolution of (Hf_0.2_Zr_0.2_Ta_0.2_Ti_0.2_Nb_0.2_)B_2_ during the tensile loading along the *b*-axis at 900 K over the strain interval of *ε* = 0.07–0.105.; Movie S2: Local structural evolution of (Hf_0.2_Zr_0.2_Ta_0.2_Ti_0.2_Nb_0.2_)B_2_ during the tensile loading along the *b*-axis at 2100 K over the strain interval of *ε* = 0.07–0.105.
