# Supplementary material for: Deep-Learning Enabled Atomistic Understanding of Thermomechanical Behaviors and Fracture Mechanisms of High-Entropy Diboride (Hf0.2Zr0.2Ta0.2Ti0.2Nb0.2)B2
Source: Materials (Basel). 2026 Jul 1;19(13):2785. doi: 10.3390/ma19132785 (PMC13362562; doi:10.3390/ma19132785)
Supplement: Supplementary file 1 [file materials-19-02785-s001.zip › materials-4402896-supplementary-done (1) edited.pdf]

materials

Supporting Information for

Deep-learning enabled atomistic understanding of thermomechanical behaviors and fracture mechanisms of high-entropy diboride $(Hf_{0.2}Zr_{0.2}Ta_{0.2}Ti_{0.2}Nb_{0.2})B_2$

Xu Zhang [a, b], Bei Li [a, b, *], Meng Wang [a, b], Bo Liu [b, c], Ji Zou [b, *] and Jianjun Li [d]

[a] School of Materials Science and Engineering, Research Center for Materials Genome Engineering, Wuhan University of Technology, Wuhan 430070, China
[b] State Key Laboratory of Advanced Technology for Materials Synthesis and Processing, Wuhan University of Technology, Wuhan 430070, China
[c] International School of Materials Science and Engineering, Wuhan University of Technology, Wuhan 430070, China
[d] State Key Laboratory of Materials Processing and Die & Mould Technology, Huazhong University of Science and Technology, Wuhan 430074, China
* Correspondence: authorE-mail addresses: libei@whut.edu.cn (B. Li); ji.zou@whut.edu.cn (J. Zou)

Supplementary Note S1: AIMD-DPMD comparison of uniaxial tension and compression

To further assess the applicability of deep learning to mechanical deformation and fracture-relevant states, we performed additional *ab initio* molecular dynamics (AIMD) calculations on $(Hf_{0.2}Zr_{0.2}Ta_{0.2}Ti_{0.2}Nb_{0.2})B_2$ under both *b*/*c*-axis tension and compression at 900 and 2100 K, which are compared with DPMD simulations, as shown in Figure S2. It is noteworthy that consistent equilibration and deformation processes were applied in both AIMD and DPMD simulations. Specifically, the $(Hf_{0.2}Zr_{0.2}Ta_{0.2}Ti_{0.2}Nb_{0.2})B_2$ system was initially equilibrated for 1 ps using the NPT ensemble with fixed box angles, and then further equilibrated for another 1 ps using the NVT ensemble. Subsequently, the tensile or compressive deformation along the *b*/*c*-axis was conducted using a stepwise strain-relaxation procedure. In each step, a strain increment of 0.02 was applied, followed by a 1 ps NPT equilibration run with fixed box angles and constant lattice dimensions along the loading direction. Stress values were averaged over the last 100 steps with a time step of 1.0 fs during each 0.02-strain substep.

Supplementary Note S2: Calculation of the fractions of under-coordinated B atoms ($f_{B\text{-}B}$ and $f_{B\text{-}M}$) and the normalized largest void-cluster volume (V_{max}^{void}/V_{box})

To provide quantitative support for the deformation and fracture mechanisms in Section 3.3.3, we introduced three structural parameters, i.e., the fractions of B atoms ($f_{B\text{-}B}$ and $f_{B\text{-}M}$) with reduced B-B and B-M coordination and the normalized largest void-cluster volume (V_{max}^{void}/V_{box}), as presented in Figure S3. Prior to estimating $f_{B\text{-}B}$ and $f_{B\text{-}M}$, critical cutoffs (i.e., $r_{cut}^{B\text{-}B}$ and $r_{cut}^{B\text{-}M}$) were defined as the first-neighbor distances in the radial distribution function profiles of B-B and B-M atomic pairs of the starting, undeformed structure, as shown in Figure S4. Then, they were set to $r_{cut}^{B\text{-}B}$ = 2.45 Å and $r_{cut}^{B\text{-}M}$ = 3.25 Å during the entire deformation process. For each B atom i, the B-B and B-M coordination numbers are calculated using

$$CN_{B\text{-}B}(i)=\sum_{j\in B, j\neq i} \mathbf{1}\left(r_{ij}\leq r_{cut}^{B\text{-}B}\right) \tag{S1}$$

$$CN_{B\text{-}M}(i)=\sum_{j\in M} \mathbf{1}\left(r_{ij}\leq r_{cut}^{B\text{-}M}\right) \tag{S2}$$

where $\mathbf{1}(\cdot)$ is the indicator function. The B-B and B-M coordination numbers for undeformed structures are $CN_{B\text{-}B}^{0} \approx 3$ and $CN_{B\text{-}M}^{0} \approx 6$, consistent with the AlB_2-type phase. For deformed structures, the B atom is identified as having reduced B-B coordination when $CN_{B\text{-}B}(i) < CN_{B\text{-}B}^{0}$, while reduced B-M coordination is identified using a stricter criterion $CN_{B\text{-}M}(i) < CN_{B\text{-}M}^{0}\text{-}1$ to prevent thermally induced neighbor fluctuations from being misclassified as coordination degradation. The corresponding under-coordination fractions are subsequently calculated using

$$f_{B\text{-}B}=\frac{\sum_{i\in B}\mathbf{1}\left[CN_{B\text{-}B}(i)<CN_{B\text{-}B}^{0}\right]}{N_B} \tag{S3}$$

$$f_{B\text{-}M}=\frac{\sum_{i\in B}\mathbf{1}\left[CN_{B\text{-}M}(i)<CN_{B\text{-}M}^{0}\text{-}1\right]}{N_B} \tag{S4}$$

Here, $f_{B\text{-}B}$ quantifies the degradation of the intralayer B-B network, and $f_{B\text{-}M}$ reflects the weakening of the local B-M coordination environment around B atoms.

For the void-cluster analysis, the instantaneous simulation box was divided into a uniform voxel grid with a voxel size of 0.75 Å. The total number of voxels is $N_{grid}=N_x N_y N_z$, and the voxel volume is calculated as $V_{voxel} = V_{box}/N_{grid}$, where V_{box} is the volume of the simulation box. A voxel is treated as occupied if its center is within a cutoff radius r_{cut} from any atom i using the minimum-image convention [S1,S2].

$$\min_i |\mathbf{r}_{voxel} - \mathbf{r}_i| \leq r_{cut} \tag{S5}$$

where $r_{cut} = \min(r_{cut}^{B\text{-}B}, r_{cut}^{B\text{-}M})$. The standard 26-neighbor connected-component analysis [S3,S4] was then performed on the unoccupied voxels to identify void clusters. For the k-th void cluster, the volume is calculated as $V_k^{void} = N_k^{void} V_{voxel}$, where N_k^{void} is the number of unoccupied voxels. The normalized largest void-cluster volume is thus obtained using

$$\frac{V_{max}^{void}}{V_{box}} = \frac{\max_k V_k^{void}}{V_{box}} = \frac{\max_k N_k^{void}}{N_{grid}} \tag{S6}$$

It is noted that V_{max}^{void}/V_{box} captures the sufficiently large and spatially connected void/crack-like damage regions. Moreover, a near-zero value indicates the absence of a percolating void cluster, rather than a complete void of local free volume.

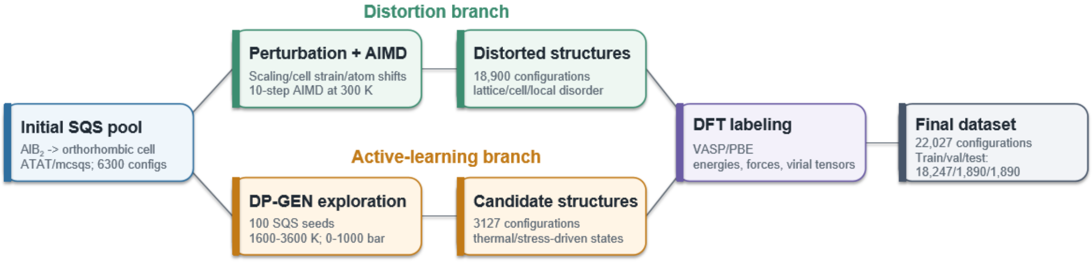

Figure S1. Workflow of dataset preparation for assessing deep learning potential.

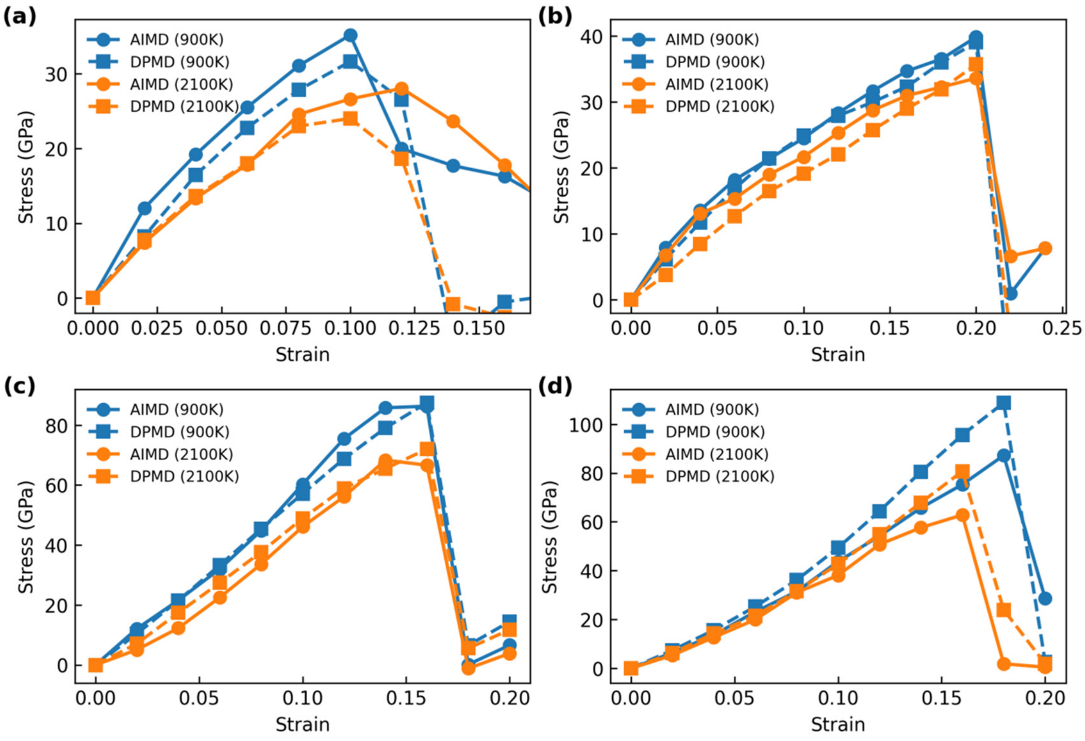

Figure S2. Comparison of stress–strain curves under different loading modes: (a) tension along the *b*-axis, (b) tension along the *c*-axis, (c) compression along the *b*-axis, and (d) compression along the *c*-axis.

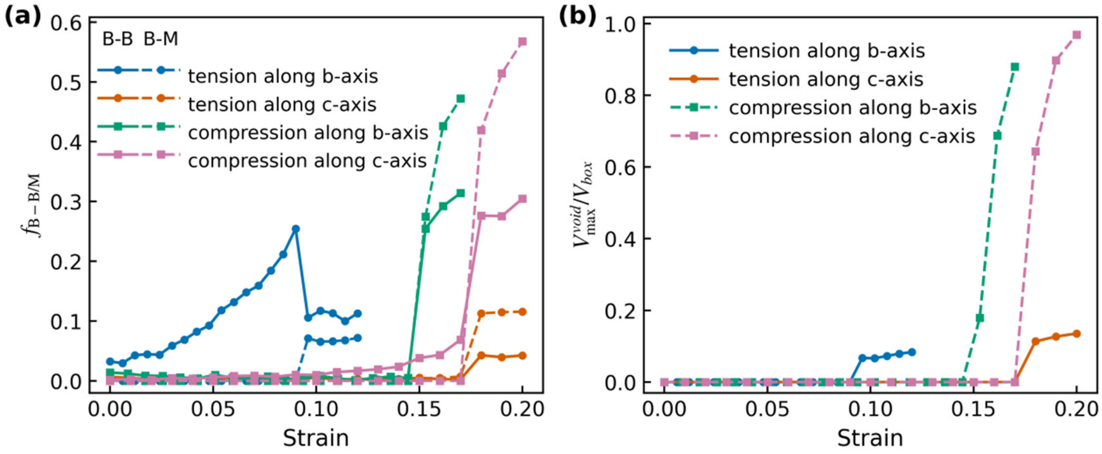

Figure S3. Evolution of the structural parameters as a function of strain during tensile and compressive loading along the *b*- and *c*-axes: (a) the fractions of under-coordinated B atoms reflecting B-B and M-B bond-network degradation, and (b) the normalized largest void-cluster volume V_{max}^{void}/V_{box}.

.

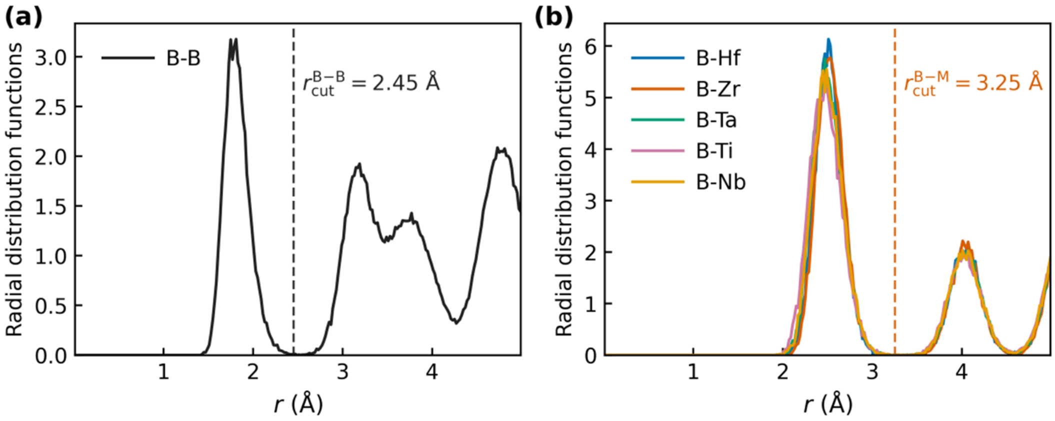

Figure S4. Radial distribution function profiles of the starting, undeformed structure: (a) B-B pairs and (b) B-M pairs.

Movie S1. Local structural evolution of $(Hf_{0.2}Zr_{0.2}Ta_{0.2}Ti_{0.2}Nb_{0.2})B_2$ during tensile loading along the *b*-axis at 900 K over the strain interval of ε = 0.07–0.105. The animation contains 701 frames and is played at 10 frames/s to show the continuous evolution of the local bonding environment and deformation process.

Movie S2. Local structural evolution of $(Hf_{0.2}Zr_{0.2}Ta_{0.2}Ti_{0.2}Nb_{0.2})B_2$ during tensile loading along the *b*-axis at 2100 K over the strain interval of ε = 0.07–0.105. The animation contains 701 frames and is played at 10 frames/s to show the continuous evolution of the local bonding environment and deformation process.

References

1. Allen, M.P.; Tildesley, D.J. *Computer Simulation of Liquids*; Oxford University Press, 2017; ISBN 978-0-19-880319-5.
2. Viscardy, S.; Gaspard, P. Viscosity in Molecular Dynamics with Periodic Boundary Conditions. *Phys. Rev. E* **2003**, *68*, 041204, doi:10.1103/PhysRevE.68.041204.
3. Kong, T.Y.; Rosenfeld, A. Digital Topology: Introduction and Survey. *Computer Vision, Graphics, and Image Processing* **1989**, *48*, 357–393, doi:10.1016/0734-189X(89)90147-3.
4. Chapman, J.; Goldman, N. Quantifying the Atomistic Free-Volume Morphology of Materials with Graph Theory. *Computational Materials Science* **2022**, *213*, 111623, doi:10.1016/j.commatsci.2022.111623.
